# Association between the atherogenic index of plasma and bone mineral density among adult women: NHANES (2011–2018)

**DOI:** 10.3389/fendo.2024.1363889

**Published:** 2024-05-21

**Authors:** Qiwang He, Bo Chen, Fuchao Liang, Zhiwen Zhang

**Affiliations:** ^1^ College of Acupuncture and Orthopedics, Hubei University of Chinese Medicine, Wuhan, China; ^2^ Department of Orthopedics, Hubei University Affiliated Hospital of Hubei University of Chinese Medicine, Wuhan, China; ^3^ Department of Endocrinology, Xiangyang Central Hospital, Affiliated Hospital of Hubei University of Arts and Science, Xiangyang, China; ^4^ Center for Clinical Evidence-Based and Translational Medicine, Xiangyang Central Hospital, Affiliated Hospital of Hubei University of Arts and Science, Xiangyang, China; ^5^ Department of Urology, Xiangyang Central Hospital, Affiliated Hospital of Hubei University of Arts and Science, Xiangyang, China

**Keywords:** atherogenic index of plasma, bone mineral density, NHANES, cross-sectional study, women

## Abstract

**Background:**

Studies on the relationship between the atherogenic index of plasma (AIP) and bone mineral density (BMD) among adult women in the United States are limited. The purpose of this study was to explore this association using a sizable, nationally representative sample.

**Methods:**

Data from the 2011 to 2018 National Health and Nutrition Examination Survey (NHANES) were used in this observational study. The AIP was computed as log_10_ (triglycerides/high-density lipoprotein cholesterol). Total BMD was measured via dual-energy X-ray densitometry. We constructed multiple linear regression models to evaluate the correlation between the AIP and BMD. The non-linear relationship was characterized by smooth curve fitting and generalized additive models. We also conducted subgroup and interaction analyses.

**Results:**

In this study, we included 2,362 adult women with a mean age of 38.13 ± 12.42 years. The results of multiple linear regression analysis, the AIP and total BMD showed a negative association (β = −0.021, 95%CI: −0.037, −0.006). The curve fitting analysis and threshold effect analysis showed a non-linear relationship between the two variables, and the inflection point of the AIP was found to be -0.61. The total BMD decreased significantly when the AIP reached this value (β = −0.03, 95%CI: −0.04, −0.01). The results of the subgroup analysis showed that AIP and total BMD had a strong negative relationship in participants who were below 45 years old (β = -0.023; 95% CI: -0.041, -0.004), overweight (BMI ≥ 25 kg/m^2^) (β = -0.022; 95% CI: -0.041, -0.002), had a higher education level (β = -0.025; 95% CI: -0.044, -0.006), and had no partners (β = -0.014; 95% CI: -0.06, -0.009).

**Conclusions:**

We found a negative correlation between the AIP and total BMD. Clinicians should pay attention to patients with high AIP, which might indicate a low BMD and has reference significance in preventing osteoporosis.

## Introduction

As the global aging problem becomes more and more serious, osteoporosis (OP), the most prevalent metabolic bone disease, has become one of the major public health problems (1, 2). An estimated 1.5 million fractures are caused by OP each year in the United States (3, 4). The economic burden of treating osteoporotic fractures is expected to reach nearly $50 billion by 2040, putting tremendous strain on American society (5, 6). Currently, the gold standard for diagnosing OP is to assess a patient’s bone mineral density (BMD) (7, 8). Therefore, it is important to identify modifiable risk factors associated with low BMD for the prevention of OP (9, 10).

Dobiásová and Frohlich introduced the atherogenic index of plasma (AIP) in 2001 as a novel lipid marker, indicating the nature and extent of aberrant lipid metabolism (11, 12). It is computed as the ratio of triglycerides (TG) with a logarithmic base of 10 to high-density lipoprotein cholesterol (HDL-C). TG, the most abundant lipid in human adipose tissue; HDL-C, contains hundreds of lipids and proteins, and a number of clinical studies have shown an association between TG and HDL-C and OP (13, 14). AIP, which combines TG and HDL-C levels, besides showing the ratio of TG to HDL-C, it also shows the particle size of lipoprotein, which is a more accurate indicator of the specificity and pathogenicity of dyslipidemia (15, 16). Several studies have shown that the AIP is a reliable indicator of cardiovascular events and death due to such events (17–19).

There is growing evidence that there is a biological link between lipid and bone metabolism, and that the disturbance of lipid metabolism can directly affect bone formation and absorption, thereby affecting the strength of bone (20, 21). Only a few epidemiological studies have investigated the relationship between AIP and BMD in the population, and these studies have found an inverse association (22–24). On the one hand, existing studies are limited to special postmenopausal population, and the association of the whole female population is not clear; on the other hand, the nonlinear association between AIP and BMD has not been deeply explored to find out the threshold of action, which is of great significance for clinical application. Meanwhile, here is a lack of studies on the association between AIP and BMD in adult women in the United States. It is estimated that approximately 40% of white women in the United States will experience at least one clinically significant osteoporotic fracture in their lifetime (25, 26). However, existing studies have focused on low and middle-income countries, and a multi-country cohort study found that among high-income countries, women in the United States had a higher risk of fracture than women in Australia, Canada, and Europe (27).

Therefore, in this study, we addressed these knowledge gaps by using the extensive National Health and Nutrition Examination Survey (NHANES) dataset and conducted an extensive cross-sectional investigation to assess the relationship between AIP and BMD among American adult women. We hypothesized that AIP and BMD are negatively correlated.

## Materials and methods

### Survey description

The multistage, cross-sectional NHANES is a nationally representative study designed to analyze the variables at risk related to health and nutrition in the American population. The NHANES started in 1999, it is performed every two years, with a new group of participants was included in each iteration of the survey. Mobile medical examination and in-home interviews are performed for the evaluation (28). The NHANES is authorized by the National Centre for Health Statistics study ethical review board, and written consent is provided by all participants (29).

### Study population

**Figure 1:**
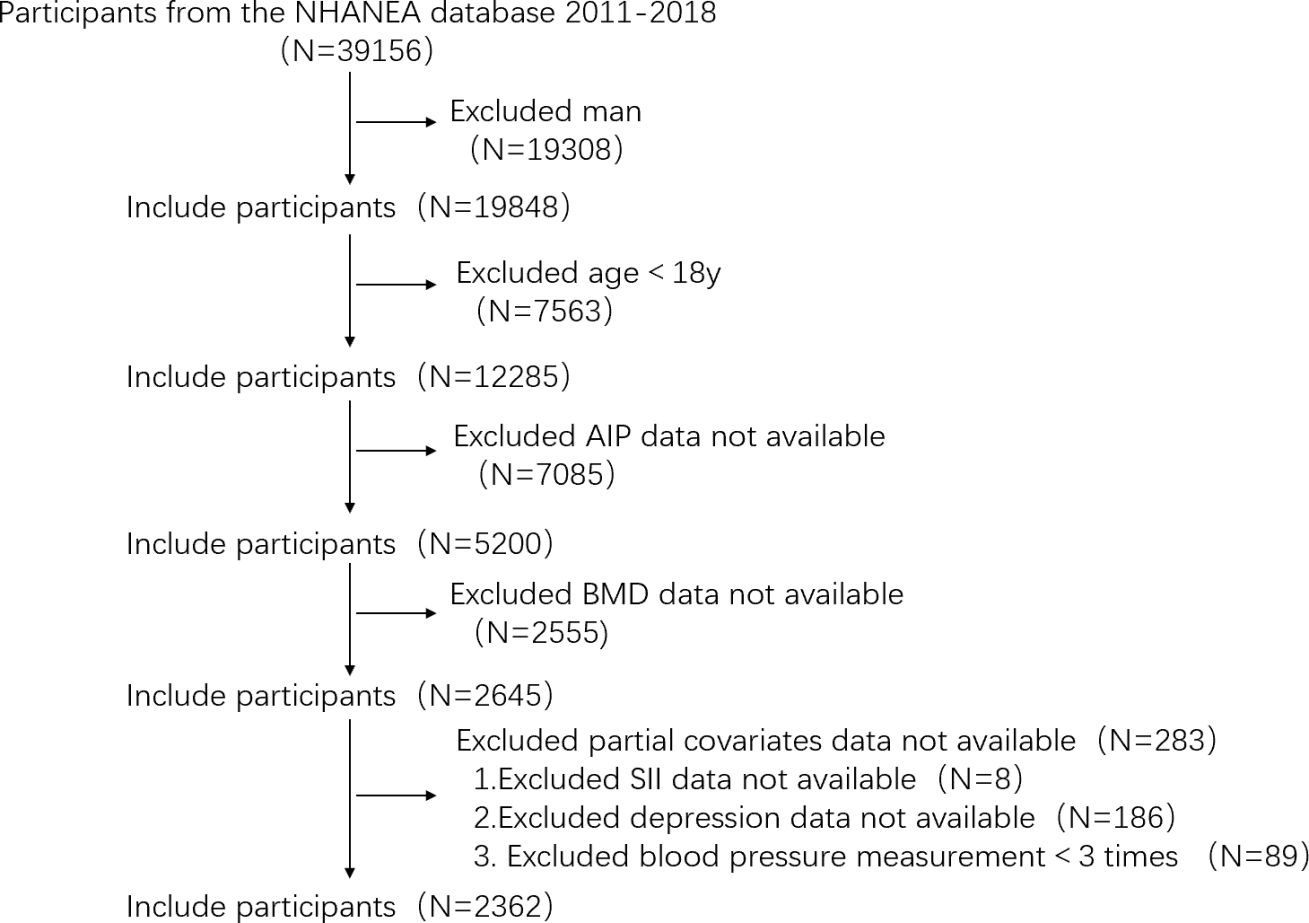
Flowchart of the sample selection process from the NHANES (2011–2018). (NHANES, National Health and Nutrition Examination Survey; AIP, atherogenic index of plasma; BMD, bone mineral density; SII, systemic immune-inflammation index).

Data collected for four two-year periods (2011–2012,2013–2014,2015–2016,2017–2018), in total, 39,156 people participated in the health examination survey. We used certain exclusion standards in our investigation to improve the reliability and validity of our conclusions. The exclusion criteria were as follows: (1) males, (2) younger than 18 years old, (3) missing AIP and total BMD data, (4) missing data for certain factors have missing data (SII, depression, blood pressure measured less than three times). In total, 2,362 individuals were included in the study (**Figure 1**).

### Atherogenic index of plasma

The AIP was defined as log_10_ (triglyceride/high-density lipoprotein cholesterol) ratio (30). Based on the AIP quartiles, all individuals were divided into four groups: group Q1 (<-0.40), group Q2 (-0.40 to <-0.20), group Q3 (-0.20 to <0.03), and group Q4 (≥0.03).

### Total bone mineral density

Previous studies have shown that the mean total BMD of non-Hispanic white women between the ages of 20 and 29 can be used as the reference value (31, 32). Any individual with BMD score of 2.5 standard deviations or more below the norm were considered osteoporosis, individuals with all BMD values of 1.0 standard deviations or more above the norm were considered normal BMD, and other cases were considered osteopenia. Finally, we collectively referred to subjects with osteoporosis or osteopenia as having a low BMD. Details are listed in **Supplementary Table S1**. Complete BMD was evaluated by DXA for all participants (included in the final analysis). The examination was performed by trained radiology technicians using Hologic QDR-4500A fan-beam densitometers (Hologic; Bedford, MA, USA). Details about the DXA exam were provided on the NHANES website (33).

### Covariates

The selection of potential BMD confounders, such as age, ratio of family income to poverty (PIR), body mass index (BMI), systolic blood pressure, diastolic blood pressure, glycohemoglobin, low-density lipoprotein cholesterol (LDL-C), total cholesterol (TC), total protein intake, total calcium intake, serum 25-hydroxyvitamin D (25(OH)D), cotinine, systemic immune-inflammation index (SII), race, education level, marital status, smoking status, alcohol consumption, trouble sleeping, depressive symptoms, coronary heart disease, diabetes, kidney failure, gout and arthritis was based on previous studies.

Age (years), PIR, BMI (kg/m^2^), systolic blood pressure (mmHg), diastolic blood pressure (mmHg), glycohemoglobin、LDL-C (mmol/L), TC (mmol/L), total protein intake (gm/d), total calcium intake (mg/d), serum 25(OH)D (nmol/L), cotinine (ng/mL), insulin (pmol/L), and SII were used as continuous variables. The average value of three measurements was recorded as the diastolic and systolic blood pressure. The SII was determined by evaluating the platelet count × neutrophil count/lymphocyte count, based on the findings of another study (34). Age (<45; ≥45 years) (35) and BMI (<25; ≥25 kg/m^2^) (36) were divided into two groups for subsequent subgroup analysis. Detailed information on the categorical variables was as follows: the education level was divided into three categories: under high school, high school or equivalent, and above high school (37). Marital status was divided into two categories:having a partner (Married/Living with a partner) and having no partner (Divorced/Never married/Widowed/Separated) (38). Individuals were categorized as smokers or non-smokers based on their answers to the question, “Have you smoked at least 100 cigarettes in your life” (39). Alcohol consumption was categorized based on the answer to the question “On days of alcohol consumption in the past 12 months, how many drinks were consumed per day on average”, and the individuals were categorized into two groups: ≥3 cups and < 3 cups per day (12). Trouble sleeping was classified as yes or no based on whether the patients told their doctor about sleeping difficulties. The instrument used to assess the symptoms of depression was the Patient Health Questionnaire (PHQ-9), scores ≥10 indicated the existence of clinically significant symptoms, and scores < 10 indicated no clinically relevant symptoms (40). Coronary heart disease, diabetes, kidney failure, gout, and arthritis were all classified as “yes” or “no” based on whether the individuals knew they had the disease.

### Statistical analysis

The AIP was partitioned into quartiles, with the reference group being the lowest quartile (Q1). Continuous variables were presented as the mean (SD) or median (IQR), whereas, categorical variables were presented as frequencies and percentages. We compared categorical and continuous variables between groups using the Chi-square test or the Fisher’s test and the one-way ANOVA test or the Kruskal-Wallis test, respectively. Multiple linear regression models were constructed to assess the link between the AIP and total BMD; smooth curve fitting and generalized additive models (GAM) were used to characterize the non-linear relationship between the AIP and total BMD. GAM is an extension of the Generalized Linear Model (GLM), which allows the modeling of nonlinear relations and non-parametric effects. The basic idea is to express the relationship between the dependent variable and multiple predictors as the sum of nonlinear functions, and to link the predictor to the response variable through the connection function, and the model parameters are estimated using maximum likelihood estimation or other appropriate methods. In model 1, the covariates were not adjusted. In model 2, age, race and BMI were adjusted. In model 3, age, race, BMI, PIR, systolic blood pressure, diastolic blood pressure, glycohemoglobin, total protein intake, total calcium intake, serum 25(OH)D, insulin, SII, education level, marital status, smoking status, trouble sleeping, depression, coronary heart disease, diabetes, kidney failure, gout and arthritis were adjusted. A subgroup analysis was conducted with stratified factors, including age (<45; ≥45 years), BMI (<25; ≥25 kg/m^2^), education level (less than high school, high school or more than high school) and marital status (having a partner or with no partner). Further, sensitivity analysis was carried out: 1. explore the relationship between BMD of femoral neck and AIP; 2. explore the relationship between OP and AIP; 3. explore the relationship between low BMD and AIP. The SPSS (version 27.0.1) software and the R programming language (version 4.3.2) were used to conduct all statistical analyses. A P-value less than 0.05 was considered to indicate statistical significance in all two-sided statistical tests.

## Results

### Baseline characteristics of the study population

In this study, 2,362 individuals (18 to 59 years old) were included. The average age of the population was 38.13 ± 12.42 years, and the mean total BMD was 1.08 ± 0.10 g/cm^2^. The clinical characteristics of the individuals according to the AIP quartiles are shown in **Table 1**. No statistically significant associations were found between marital status, coronary heart disease, kidney failure, total protein intake, total calcium intake, serum 25(OH)D and the AIP (P>0.05). Individuals with AIP levels in the upper quartile were more likely to be non-Hispanic black (40.8%), have a high school education or above (54.5%), be overweight (BMI 32.43 ± 7.12), and have a lower family income-to-poverty ratio (2.20 ± 1.58). With the increase of AIP level, the proportion of smokers, patients with ≥3 cups of alcohol intake, patients with sleep difficulties, patients with depression, patients with diabetes, patients with gout and arthritis showed an increasing trend. Systolic blood pressure, diastolic blood pressure, glycohemoglobin, LDL-C, TC, cotinine, insulin, and SII were highest at Q4 levels compared to the low quartile of AIP.

**Table 1:** Baseline characteristics of the study population based on the AIP quartiles.

Variable	Total	Q1 (<-0.40)	Q2 (-0.40 to <-0.20)	Q3 (-0.20 to <0.03)	Q4 (≥0.03)	*P*
Race (%)						<0.001
Mexican American	354 (15.0)	55 (9.3)	74 (12.5)	100 (16.9)	125 (21.2)	
Other Hispanic	254 (10.8)	51 (8.7)	57 (9.6)	74 (12.5)	72 (12.2)	
Non-Hispanic black	839 (35.5)	188 (31.9)	202 (34.1)	208 (35.2)	241 (40.8)	
Non-Hispanic white	519 (22.0)	179 (30.4)	158 (26.7)	120 (20.3)	62 (10.5)	
Other race	396 (16.8)	116 (19.7)	101 (17.1)	89 (15.1)	90 (15.3)	
Education level (%)						<0.001
Under high school	358 (16.4)	48 (9.2)	61 (11.1)	107 (19.7)	142 (24.8)	
High school or equivalent	419 (19.2)	69 (13.2)	104 (19.0)	127 (23.4)	119 (20.8)	
Above high school	1410 (64.5)	407 (77.7)	383 (69.9)	308 (56.8)	312 (54.5)	
Marital status (%)						0.140
Having a partner	1253 (57.3)	295 (56.3)	304 (55.5)	309 (57.0)	345 (60.2)	
No partner	934 (42.7)	229 (43.7)	244 (44.5)	233 (43.0)	228 (39.8)	
Smoking status (%)						<0.001
Yes	739 (31.8)	141 (24.4)	162 (27.7)	190 (33.2)	246 (41.8)	
No	1583 (68.2)	437 (75.6)	422 (72.3)	382 (66.8)	342 (58.2)	
Alcohol consumption (%)						0.019
≥3	492 (29.9)	109 (25.1)	132 (29.9)	130 (33.5)	121 (41.8)	
<3	1151 (70.1)	326 (74.9)	310 (70.1)	258 (66.5)	257 (68.0)	
Trouble sleeping (%)						<0.001
Yes	634 (26.8)	125 (21.2)	132 (22.3)	167 (28.3)	210 (35.6)	
No	1728 (73.2)	464 (78.8)	460 (77.7)	424 (71.7)	380 (64.4)	
Depressive symptoms (%)						<0.001
Yes	233 (9.9)	36 (6.1)	45 (7.6)	57 (9.6)	95 (16.1)	
No	2129 (90.1)	553 (93.3)	547 (92.4)	534 (90.4)	495 (83.9)	
Coronary heart disease (%)						0.618
Yes	13 (0.6)	3 (0.6)	1 (0.2)	2 (0.4)	7 (1.2)	
No	2173 (99.4)	521 (99.4)	547 (99.8)	540 (99.6)	565 (98.8)	
Diabetes (%)						<0.001
Yes	175 (7.6)	10 (1.7)	21 (3.6)	37 (6.4)	107 (18.6)	
No	2138 (92.4)	569 (98.3)	557 (96.4)	545 (93.6)	467 (81.4)	
Kidney failure (%)						0.142
Yes	51 (2.3)	5 (1.0)	8 (1.5)	16 (3.0)	22 (3.8)	
No	2134 (97.7)	519 (99.0)	539 (98.5)	526 (97.0)	550 (96.2)	
Gout (%)						<0.001
Yes	34 (1.6)	3 (0.6)	7 (1.3)	4 (0.7)	20 (3.5)	
No	2153 (98.4)	521 (99.4)	541 (98.7)	538 (99.3)	553 (96.5)	
Arthritis (%)						<0.001
Yes	378 (17.3)	47 (9.0)	85 (15.5)	95 (17.6)	151 (26.5)	
No	1803 (82.7)	476 (91.0)	462 (84.5)	445 (82.4)	420 (73.5)	
Age (year)	38.13 ± 12.42	35.16 ± 12.11	37.10 ± 12.36	38.90 ± 12.76	41.37 ± 11.58	<0.001
PIR	2.41 ± 1.65	2.67 ± 1.69	2.52 ± 1.66	2.23 ± 1.64	2.20 ± 1.58	<0.001
BMI (kg/m^2^)	29.19 ± 7.76	25.68 ± 6.78	28.15 ± 7.64	30.51 ± 7.76	32.43 ± 7.12	<0.001
Systolic blood pressure (mmHg)	116.01 ± 15.15	112.68 ± 14.55	114.53 ± 14.25	116.62 ± 15.60	120.21 ± 15.15	<0.001
Diastolic blood pressure (mmHg)	69.35 ± 10.42	67.64 ± 10.54	68.55 ± 9.86	69.28 ± 10.68	71.95 ± 10.09	<0.001
Glycohemoglobin (%)	5.59 ± 0.99	5.31 ± 0.51	5.45 ± 0.79	5.58 ± 0.93	6.02 ± 1.39	<0.001
LDL-C (mmol/L)	2.87 ± 0.88	2.49 ± 0.74	2.81 ± 0.79	3.00 ± 0.85	3.17 ± 0.99	<0.001
TC (mmol/L)	4.87 ± 1.02	4.58 ± 0.91	4.73 ± 0.94	4.90 ± 0.97	5.27 ± 1.11	<0.001
Total protein intake (gm/d)	67.18 (48.26,90.67)	68.11 (51.13,94.51)	69.82 (49.16,92.46)	64.78 (45.78,85.92)	66.41 (46.38,90.31)	0.059
Total calcium intake (mg/d)	736.00 (491.00,1073.00)	737.00 (492.00,1087.75)	768.00 (514.00,1083.00)	710.50 (482.00,1051.50)	739.00 (481.75,1061.50)	0.977
Serum 25(OH)D (nmol/L)	58.80 (43.10,77.40)	59.30 (41.75,78.35)	59.00 (42.50,75.95)	58.10 (43.30,77.90)	58.95 (44.05,77.93)	0.695
Cotinine (ng/mL)	0.03 (0.01,1.26)	0.03 (0.01,0.18)	0.03 (0.01,0.74)	0.04 (0.01,5.92)	0.04 (0.01,81.95)	<0.001
Insulin (pmol/L)	56.97 (36.17,90.56)	37.44 (25.20,53.97)	52.62 (36.06,76.38)	63.90 (40.86, 94.20)	90.90 (57.42,137.94)	<0.001
SII	452.06 (324.22,632.00)	399.64 (290.47,568.89)	426.56 (314.66,598.52)	488.84 (667.80,341.00)	504.57 (360.77,675.39)	<0.001
Total BMD (g/cm^2^)	1.08 ± 0.10	1.09 ± 0.97	1.09 ± 0.10	1.08 ± 0.10	1.07 ± 0.10	<0.001

The mean (SD) or median (IQR) values of continuous variables, and the p-value was calculated by the one-way ANOVA test or the Kruskal–Wallis test. Percentage for categorical variables, the p-value was calculated by the Chi-square test or the Fisher’s test. (AIP, atherogenic index of plasma; PIR, ratio of family income to poverty; BMI, body mass index; LDL-C, low-density lipoprotein cholesterol; TC, total cholesterol;25(OH)D, 25-hydroxyvitamin D;SII, systemic immune-inflammation index; BMD, bone mineral density).

### Relationship between the AIP and BMD

Multiple linear regression analysis was performed to assess the relationship in three model between the AIP and total BMD in the three models. In model 1, no covariates were adjusted. In model 2, adjustments were made for age, race, and BMI. In model 3, further adjustments were made for PIR, systolic blood pressure, diastolic blood pressure, glycohemoglobin, total protein intake, total calcium intake, serum 25(OH)D, SII, education level, marital status, smoking status, trouble sleeping, depressive symptoms, coronary heart disease, diabetes, kidney failure, gout and arthritis (**Table 2**). The AIP and total BMD were found to have a negative relationship (β = -0.022; 95% CI: -0.034, -0.009). After adjusting for confounders, this negative correlation was found in model 2 (β = −0.018; 95%CI: −0.03, −0.005) and model 3 (β = −0.021; 95%CI: −0.037, −0.006) was still present. The AIP was converted into a categorical variable (quartile) from a continuous variable. The trend test was significant (P for trend < 0.001) in all three models. In Model 3, the total BMD of the highest quartile was 0.02g/cm² lower than that of the lowest quartile.

**Table 2:** Associations between the AIP and total BMD.

	Model 1			Model 2			Model 3		
	β	95% CI	P	β	95% CI	P	β	95% CI	P
AIP	-0.022	-0.034, -0.009	<0.001	-0.018	-0.030, -0.005	<0.01	-0.021	-0.037, -0.006	<0.01
AIP(Quartile)									
Q1	Reference			Reference			Reference		
Q2	0	-0.011, 0.011	0.988	-0.002	-0.013, 0.008	0.679	0	-0.012, 0.012	0.994
Q3	-0.011	-0.022, 0.001	0.067	-0.013	-0.024, -0.002	<0.05	-0.008	-0.021, 0.004	0.188
Q4	-0.021	-0.033, -0.01	<0.001	-0.019	-0.031, -0.008	<0.001	-0.020	-0.034, -0.007	<0.01
P for trend	<0.001			<0.001			<0.001		

AIP: Q1 (<-0.40), Q2 (-0.40 to<-0.20), Q3 (-0.20 to <0.03), and Q4 (≥0.03); (AIP, atherogenic index of plasma; BMD, bone mineral density; BMI, body mass index; PIR, ratio of family income to poverty; 25(OH)D, 25-hydroxyvitamin D; SII, systemic immune-inflammation index; CI, confidence interval).

Model 1 no parameter was adjusted.

Model 2 continuous variables (age and BMI), categorical variables (race).

Model 3 continuous variables (age, BMI, PIR, systolic blood pressure, diastolic blood pressure, glycohemoglobin, total protein intake, total calcium intake, serum 25(OH)D, insulin, and SII), categorical variables (race, educational level, marital status, smoking status, trouble sleeping, depressive symptoms, coronary heart disease, diabetes, kidney failure, gout, and arthritis).

### The non-linear relationship between the AIP and BMD

**Figure 2:**
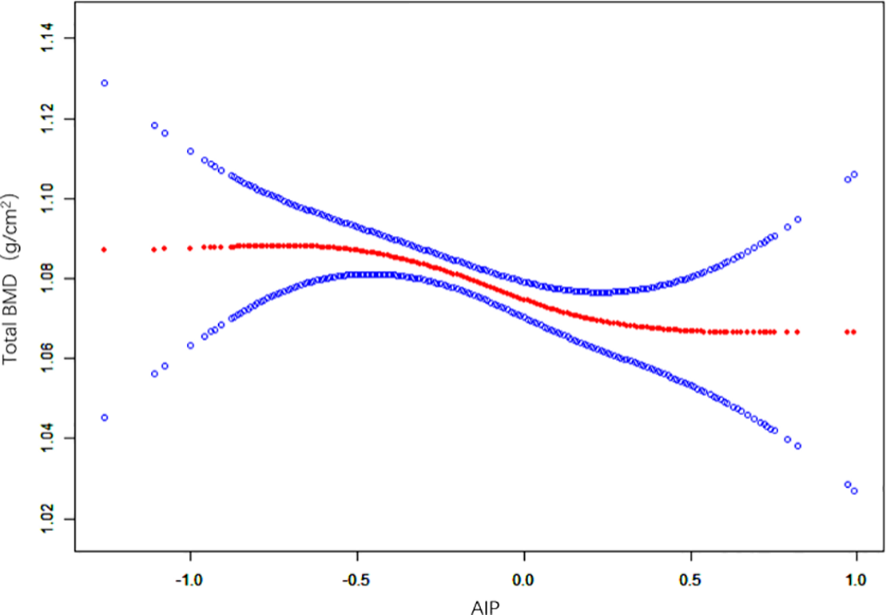
Non-linear relationship between the AIP and total BMD. (AIP, atherogenic index of plasma; BMD, bone mineral density).

The non-linear relationship between the AIP and total BMD is shown in **Figure 2**. Using the two-segment piecewise linear regression model, we found that -0.61 was the AIP turning point. When the AIP was lower than -0.61, no difference was found in the total BMD with an increase in AIP, and the β value was 0.06 (95%CI: -0.07, 0.19). When the AIP was ≥ -0.61, it was negatively correlated with BMD, and the β value was -0.03 (95%CI: -0.04, -0.01), as shown in **Table 3**.

**Table 3:** Threshold effect analysis of the AIP and total BMD using the two-segment piecewise linear regression model.

Total BMD	Adjusted β (95% CI) P value
AIP	
Inflection point	-0.61
AIP < Inflection point	0.06 (-0.07, 0.19) 0.387
AIP > Inflection point	-0.03 (-0.04, -0.01) 0.003
Log-likelihood ratio	0.039

Age, race, BMI, PIR, systolic blood pressure, diastolic blood pressure, glycohemoglobin, total protein intake, total calcium intake, serum 25(OH)D, SII, education level, marital status, smoking status, trouble sleeping, depressive symptoms, coronary heart disease, diabetes, kidney failure, gout, and arthritis were adjusted. (AIP, atherogenic index of plasma; BMD, bone mineral density; CI, confidence interval).

### Subgroup analysis and interaction test

A subgroup analysis was performed to estimate the relationship between the AIP and total BMD (**Table 4**). A negative association between the AIP and total BMD was recorded in participants who were below 45 years old (β = -0.023; 95% CI: -0.041, -0.004), overweight (β = -0.022; 95% CI: -0.041, -0.002), with a higher education level (β = -0.025; 95% CI: -0.044, -0.006) and with no partners (β = -0.014; 95% CI: -0.06, -0.009) was more obvious. Strong interactions were found among BMI, education level and marital status.

**Table 4:** Subgroup analysis for the association between the AIP and total BMD.

Subgroup analysis	β	95% CI	P-value	P for interaction
Age (year)				0.056
<45	-0.023	-0.041, -0.004	<0.05	
≥45	-0.022	-0.051, 0.006	0.127	
BMI (kg/m^2^)				<0.05
<25	-0.013	-0.043, 0.016	0.378	
≥25	-0.022	-0.041, -0.002	<0.05	
Education level				<0.01
Less than high school	-0.009	-0.052, 0.034	0.685	
High school	-0.007	-0.045, 0.031	0.730	
More than high school	-0.025	-0.044, -0.006	<0.05	
Marital status				<0.01
Having a partner	-0.010	-0.030, 0.010	0.314	
No partner	-0.034	-0.060, -0.009	<0.01	

(AIP, atherogenic index of plasma; BMD, bone mineral density; BMI, body mass index; CI, confidence interval).

### Sensitivity analysis

The results of sensitivity analysis were consistent with those of main analysis. Details are listed in **Supplementary Table S2**.

## Discussion

In this study, the results of multiple regression analysis revealed a negative correlation between the AIP and total BMD. Curve fitting and threshold effect analyses revealed a non-linear relationship between the two, and the inflection point was -0.61. When the AIP was ≥-0.61, the total BMD decreased with increasing AIP. The results of the subgroup analysis showed that this association was prominent in individuals who were < 45 years, with a BMI ≥25 (kg/m^2^), a high education level, and without a partner.

Three studies were previously conducted on the AIP and BMD. Ersoy et al. found that the AIP negatively affected the BMD of postmenopausal women (22). Hernández et al. reported that the AIP was significantly and independently associated with bone microstructure degradation in Spanish women, suggesting that when evaluating postmenopausal women’s total bone metabolism, the AIP may be a helpful technique (23). In Sudanese women, Elmugadam et al. found that the AIP was positively associated with the risk of OP in postmenopausal women (24). Our findings were similar to those of previous studies. Using the NHANES database, we were the first study to show a negative relationship between the AIP and BMD among adult women in the United States. Based on these findings, the AIP is considered to be related to bone metabolism in women of different countries and ethnicities, and we hypothesize that the AIP could be used to manage and prevent OP effectively. Therefore, studies on the AIP and BMD or OP need to be performed in all populations with more participants to provide stronger evidence.

Although the common mechanism by which the AIP and bone loss develop is unclear, there are several explanations. First, adipokines such as lipocalin, leptin and chemotaxin promote the formation of atherosclerosis but they also participate in the remodeling of bones (41–44). For example, Varri et al. studied 290 postmenopausal women in Finland and found a connection between poor bone density, adipokines and vascular calcification (45). Second, systemic inflammation is also associated with levels of TG, HDL-C, and bone metabolism (46, 47). For example, Huang et al. found that HDL-C levels were lower and TG levels were higher in the group with systemic lupus erythematosus (SLE), compared to the healthy control group; Ruaro et al. found that BMD and trabecular scores were lower in SLE patients than in healthy matched controls (48, 49). Third, biological factors related to bone metabolism can also affect the levels of TG and HDL-C. For example, Sherief et al. found a positive correlation between serum osteoprotectin levels and TG; Fryes et al. found that the osteosclerosis protein had a positive correlation with TG and a negative correlated with HDL-C (50, 51). Since several clinical studies have shown that the AIP and bone metabolism are correlated, more basic studies are needed to answer the molecular mechanism of the two.

The results of the subgroup analysis showed that the AIP could predict BMD in women below 45 years of age and BMI ≥25 (kg/m^2^). Several studies have shown that age strongly influences the AIP and BMD (52, 53). Our results were inconsistent with those of previous studies that found a negative association between the AIP and BMD in postmenopausal women (22–24). Through comparison, we found that it might be caused by differences in the study design, ethnic characteristics, measurement sites, etc. Some relevant covariates such as estrogen levels were not included in this study. Second, in obese patients, the AIP was negatively correlated with BMD. Some studies have shown that, while obesity is closely related to an increase in the AIP, an increase in BMI may have adverse effects on the health of women (54–56). Although the exact process behind bone deterioration in obese individuals remains unclear, we speculated that inflammation and alterations in the hormone levels that regulate bone might influence the relationship between the AIP and BMD (57, 58).

Interestingly, we found a negative association between AIP and BMD in the higher education subgroup and no partner subgroup. Although little has been reported about this, through extensive literature review and clinical experience, we have identified several potential mechanisms to explain it. First, people with higher education levels may have higher disease awareness and take timely preventive measures and treatment (59). Second, people with higher levels of education have higher levels of income relative to those with lower levels of education. On the one hand, they have better nutrition and health during childhood and adolescence. On the other hand, they are more likely to have access to a healthy lifestyle, exercise opportunities and better health care (60, 61). For the single subgroup, first, the lifestyle of the single population (such as diet, physical activity patterns, etc.) may be relatively unhealthy compared with that of the partner population, and a single life may be more casual, and lack of care and supervision from others. Second, single people lack of sexual life, but appropriate sexual life has many benefits, such as improving sleep, reducing pain, soothing mood, etc., in the sexual process, women’s pelvic congestion, accelerate local blood circulation, to a certain extent, can promote the blood supply of the uterus and ovaries, conducive to health (62).

As a rule, the ovarian function of postmenopausal women declines, the secretion of estrogen in the body is significantly reduced, and the lack of estrogen leads to more obvious upregulation of osteoclast activity than osteoblast activity, and bone absorption accelerates and exceeds the rate of bone formation, resulting in rapid bone loss, thereby reducing BMD. On the other hand, estrogen levels also have an effect on triglyceride and HDL-C levels. Several studies have shown a negative correlation between estrogen and TG (63, 64). Some studies have found a positive correlation between estrogen and HDL-C (65, 66). In summary, covariable estrogen has a greater impact on the relationship between BMD and AIP. Therefore, the relationship between BMD and AIP in participants under 45 years old is less affected by estrogen fluctuations, which can better demonstrate the correlation between the two. Lipid metabolism is closely related to bone metabolism, and the disorder of lipid metabolism can directly affect the formation and absorption of bone, thus affecting BMD (21). A number of studies have found that cholesterol and TG are significantly correlated with BMD (67, 68). A high-cholesterol diet significantly reduced BMD and osteoblast activity, while increasing levels of bone resorption markers such as type I collagen pyridinoline cross-linked fragments (69, 70). Through experimental studies, it was found that high-cholesterol diet inhibited the proliferation and differentiation of osteoblast MC3T3-E, and after treatment, the expressions of bone morphogenetic protein (BMP2), dwarf-related transcription factor 2 (Runx2), alkaline phosphatase (ALP, ALPL), collagen type 1 (COL2A1) and other osteogenic genes were reduced. The normal expression of these genes is an important factor in the osteoblast process, suggesting that free cholesterol may inhibit the expression of Runx2, ALPL and COL2A1 in osteoblasts by inhibiting BMP2, thereby inhibiting the differentiation of osteoblasts. At the same time, a high-cholesterol diet may inhibit TGF-β/BMP2/Wnt signaling, which is essential for mammalian bone formation and is responsible for almost all osteoblast functions (71–73).

Compared to previous studies, our study had several advantages. First, previous studies on the AIP and BMD did not involve the U.S. population and were mostly limited to postmenopausal women, we were the first to investigate the connection between adult AIP and BMD in adult women from the USA. Second, we obtained data from the NHANES database, which has a relatively large sample size and excellent population representation. Third, the inflection point was found using the threshold effect analysis. However, our study has several limitations. First, this study had a cross-sectional design, which prevented us from determining the intricate causal link between the AIP and BMD. Second, as this was an observational study, we could not rule out any potential confounding factors that might have affected the outcomes. In order to improve the accuracy and authenticity of the results, we adjusted the relevant covariates available as far as possible. Third, Americans were included in this study, and thus, it is not known if the correlation between the AIP and BMD valid for people from other nations or ethnic backgrounds due to variations in genetic and environmental and other parameters. Fourth, self-report questionnaires were used to obtain information on some of the covariate data, which may not fully reflect the circumstances and may induce memory bias. Fifth, there are other methods to diagnose OP besides BMD, and more accurate studies will be conducted by combining multiple methods in the future. Therefore, given the limitations of this study, studies with a better design are needed to validate our findings.

## Conclusions

To summarize, the results of this study showed a negative correlation between the AIP and total BMD. The AIP cut-off (-0.61) has a certain clinical application value, indicating that adult women in the United States might have a low BMD, which might contribute to the prevention of osteoporosis.

## Data availability statement

Publicly available datasets were analyzed in this study. This data can be found here: Centers for Disease Control and Prevention (CDC), National Center for Health Statistics (NCHS), National Health and Nutrition Examination Survey (NHANES), https://wwwn.cdc.gov/nchs/nhanes/Default.aspx, NHANES 2011-2018.

## Ethics statement

Ethical review and approval was not required for the study on human participants in accordance with the local legislation and institutional requirements. Written informed consent from the patients/participants or patients/participants’ legal guardian/next of kin was not required to participate in this study in accordance with the national legislation and the institutional requirements.

## Author contributions

QH: Conceptualization, Data curation, Formal analysis, Investigation, Methodology, Project administration, Software, Validation, Visualization, Writing – original draft, Writing – review & editing. BC: Conceptualization, Methodology, Software, Writing – review & editing. FL: Writing – review & editing. ZZ: Funding acquisition, Project administration, Supervision, Resources, Writing – review & editing.
