# Supplementary material for: Association between the atherogenic index of plasma and bone mineral density among adult women: NHANES (2011–2018)
Source: Front Endocrinol (Lausanne). 2024 May 21;15:1363889. doi: 10.3389/fendo.2024.1363889 (PMC11148244; doi:10.3389/fendo.2024.1363889)
Supplement: Supplementary file 1 [file DataSheet_1.docx]

**Supplementary Material**

**Supplementary Table S1 Definition of osteopenia and osteoporosis**

|  | **Mean** | **SD** | **Mean-2.5SD** | **Mean-SD** | **Osteoporosis** | **Osteopenia** | **Normal BMD** |
| --- | --- | --- | --- | --- | --- | --- | --- |
| Total BMD (g/cm2) | 1.076 | 0.093 | 0.845 | 0.983 | ≤0.845 | 0.845＜ and ＜0.983 | ≥0.983 |

BMD, bone mineral density; SD, standard deviation

**Supplementary Table S2 Sensitivity analysis**

Associations between femoral neck BMD and AIP

| **Continuous or categories** | **β** | **95%CI low** | **95%CI upp** | **P-value** |
| --- | --- | --- | --- | --- |
| AIP | -0.25 | -0.069 | 0.018 | 0.25 |
| Q1 | Reference | | | |
| Q2 | -0.013 | -0.046 | 0.021 | 0.459 |
| Q3 | 0.018 | -0.016 | 0.052 | 0.295 |
| Q4 | -0.031 | -0.069 | 0.007 | 0.110 |

BMD, bone mineral density; AIP, atherogenic index of plasma; CI, confidence intervals

Association of the risk of osteoporosis with AIP

| **Continuous or categories** | **OR** | **95%CI low** | **95%CI upp** | **P-value** |
| --- | --- | --- | --- | --- |
| AIP | 0.898 | 0.104 | 7.773 | 0.922 |
| Q1 | Reference | | | |
| Q2 | 0.599 | 0.069 | 5.174 | 0.641 |
| Q3 | 0.828 | 0.116 | 5.93 | 0.851 |
| Q4 | 0.764 | 0.096 | 6.049 | 0.798 |

BMD, bone mineral density; AIP, atherogenic index of plasma; CI, confidence intervals

Association of the risk of low BMD with AIP

| **Continuous or categories** | **OR** | **95%CI low** | **95%CI upp** | **P-value** |
| --- | --- | --- | --- | --- |
| AIP | 0.796 | 0.499 | 1.268 | 0.336 |
| Q1 | Reference | | | |
| Q2 | 1.039 | 0.708 | 1.525 | 0.845 |
| Q3 | 0.81 | 0.549 | 1.195 | 0.288 |
| Q4 | 0.763 | 0.508 | 1.147 | 0.194 |

BMD, bone mineral density; AIP, atherogenic index of plasma; CI, confidence intervals
